# Draft Genome Sequence of *Microbacterium* sp. Strain Alg239_V18, an Actinobacterium Retrieved from the Marine Sponge *Spongia* sp.

**DOI:** 10.1128/genomeA.01457-16

**Published:** 2017-01-19

**Authors:** Elham Karimi, Jorge M. S. Gonçalves, Margarida Reis, Rodrigo Costa

**Affiliations:** a Center of Marine Sciences (CCMAR), Algarve University, Faro, Portugal; b Faculty of Science and Technology, Algarve University, Faro, Portugal; c Institute for Bioengineering and Biosciences (IBB), Department of Bioengineering, IST, Universidade de Lisboa, Lisbon, Portugal

## Abstract

Here, we describe the draft genome sequence of *Microbacterium* sp. strain Alg239_V18, an actinobacterium retrieved from the marine sponge *Spongia* sp. Genome annotation revealed a vast gene repertoire involved in antibiotic and heavy metal-resistance, and a versatile carbohydrate assimilation metabolism with potential for chitin utilization.

## GENOME ANNOUNCEMENT

*Microbacterium* spp. are aerobic, Gram-positive actinobacteria found in diverse environments (1)—including marine habitats such as deep-sea sediments (2, 3), seawater (4), bivalves (5), and marine sponges (6–8)—and possess the ability to produce pharmaceutically important natural products such as the bioactive compound glucosylmannosyl-glycerolipid (7). Although several *Microbacterium* genomes are publicly available, most derive from terrestrial sources and very few represent host-associated strains. *Microbacterium* spp. are among the prevalent actinobacteria cultivated from marine sponges (9, 10), but little is known about their functional features and possible roles in this interaction. To increase our understanding of the metabolic potential of these marine sponge associates, we report here the draft genome sequence of *Microbacterium* sp. strain Alg239_V18, cultivated from the marine sponge *Spongia* sp. sampled off the coast of Algarve, South Portugal. Genomic DNA of *Microbacterium* sp. strain Alg239_V18 was extracted with the Wizard genomic DNA purification kit (Promega Corporation, Madison, WI, USA) after cultivation and purification of the colony on VXA medium (double-strength VL55 medium (11) supplemented with 0.05% xylan and solidified with agar) and subsequent overnight growth in liquid VX medium. Genome sequencing was performed at Mr. DNA (Shallowater, TX, USA) on an Illumina MiSeq device using paired-end, 2 × 301-bp libraries. Sequencing depth was 0.97 Gb, leading to 298× coverage of the genome, which was assembled *de novo* into 22 *Microbacterium* contigs with the NGen DNA assembly software by DNAStar, Inc. The resulting draft genome sequence was annotated with the Rapid Annotations Using Subsystems Technology (RAST) prokaryotic genome annotation server (version 2.0) using standard procedures (12). Secondary metabolite- and antibiotic-encoding gene clusters were predicted with antiSMASH (13) and NapDos (14).

The genome is 3,228,018 bp in length, featuring a GC content of 69.4% and 3,061coding sequences, in addition to five rRNAs and 45 tRNAs. *Microbacterium* sp. strain Alg239_V18 displayed 98.8% 16S rRNA gene similarity with its closest relative, *M. aquimaris* JS54-2(T), isolated from seawater (4). Genome annotation displayed a broad range of genes possibly involved in antibiotic (e.g., fluoroquinolones) and heavy metal resistance (e.g., arsenic, cobalt, copper, and mercury); the latter observation is consistent with the recent isolation of several *Microbacterium* strains from heavy metal–contaminated soils (15). A diversified carbohydrate metabolism can be inferred from the annotated genome, encompassing multiple genes involved in the assimilation of various mono-, di-, and oligosaccharides, and 17 genes related to chitin and N-acetylglucosamine utilization. Using antiSMASH, we found a putative tetraterpene biosynthetic gene cluster displaying similar architecture to that of *M. testaceum* StLB037, in addition to one type III polyketide synthase gene cluster resembling that of *M. yannicii* PS01. Curiously, terpene classes such as furanoterpenes, furanosesterterpenes, and sesterterpenes are regularly retrieved from *Spongia officinalis*, and some display biofilm-inducing capacities (16). The search for natural product domains using NaPDoS retrieved a putative modular KS domain similar to that involved in the biosynthesis of candicidin, an antifungal compound usually obtained from the actinobacterium *Streptomyces griseus* and applied in the treatment of candidiasis.

### Accession number(s).

This draft genome sequence of* Microbacterium* sp. strain Alg239_V18 was deposited in the European Nucleotide Archive (ENA) (http://www.ebi.ac.uk/ena) under the accession numbers FMSE01000001 to FMSE01000022. The study identification number is PRJEB15584.

## References

[1] Collins M, Bradbury J 1992 The genera *Agromyces*, *Aureobacterium*, *Clavibacter*, *Curtobacterium*, and *Microbacterium*, p 1355–1368. *In* Balows A, Truper HG, Dworkin M, Harder W, Schleifer K-H (ed), The prokaryotes. Springer, New York.

[2] Shivaji S, Bhadra B, Rao RS, Chaturvedi P, Pindi PK, Raghukumar C 2007 *Microbacterium indicum* sp. nov., isolated from a deep-sea sediment sample from the Chagos Trench, Indian Ocean. Int J Syst Evol Microbiol 57:1819–1822. doi:10.1099/ijs.0.64782-0. 17684264

[3] Wu YH, Zhou P, Cheng H, Wang CS, Wu M, Xu XW 2015 Draft genome sequence of *Microbacterium profundi* Shh49^T^, an actinobacterium isolated from deep-sea sediment of a polymetallic nodule environment. Genome Announc 3(3):e00642-00615. doi:10.1128/genomeA.00642-15. 26067975 PMC4463539

[4] Kim KK, Lee KC, Oh HM, Lee JS 2008 *Microbacterium aquimaris* sp. nov., isolated from seawater. Int J Syst Evol Microbiol 58:1616–1620. doi:10.1099/ijs.0.65763-0. 18599704

[5] Chauhan A, Green S, Pathak A, Thomas J, Venkatramanan R 2013 Whole-genome sequences of five oyster-associated bacteria show potential for crude oil hydrocarbon degradation. Genome Announc 1(5):e00802-00813. doi:10.1128/genomeA.00802-13. 24092793 PMC3790097

[6] Wicke C, Hüners M, Wray V, Nimtz M, Bilitewski U, Lang S 2000 Production and structure elucidation of glycoglycerolipids from a marine sponge-associated *Microbacterium* species. J Nat Prod 63:621–626. doi:10.1021/np990313b. 10843572

[7] Lang S, Beil W, Tokuda H, Wicke C, Lurtz V 2004 Improved production of bioactive glucosylmannosyl-glycerolipid by sponge-associated *Microbacterium* species. Mar Biotechnol (NY) 6:152–156. doi:10.1007/s10126-003-0009-5. 15085410

[8] Tabares P, Pimentel-Elardo SM, Schirmeister T, Hünig T, Hentschel U 2011 Anti-protease and immunomodulatory activities of bacteria associated with Caribbean sponges. Mar Biotechnol (NY) 13:883–892. doi:10.1007/s10126-010-9349-0. 21222136 PMC7088305

[9] Abdelmohsen UR, Bayer K, Hentschel U 2014 Diversity, abundance and natural products of marine sponge-associated actinomycetes. Nat Prod Rep 31:381–399. doi:10.1039/c3np70111e. 24496105

[10] Simister RL, Deines P, Botté ES, Webster NS, Taylor MW 2012 Sponge-specific clusters revisited: a comprehensive phylogeny of sponge-associated microorganisms. Environ Microbiol 14:517–524. doi:10.1111/j.1462-2920.2011.02664.x. 22151434

[11] Sait M, Hugenholtz P, Janssen PH 2002 Cultivation of globally distributed soil bacteria from phylogenetic lineages previously only detected in cultivation-independent surveys. Environ Microbiol 4:654–666. doi:10.1046/j.1462-2920.2002.00352.x. 12460273

[12] Overbeek R, Olson R, Pusch GD, Olsen GJ, Davis JJ, Disz T, Edwards RA, Gerdes S, Parrello B, Shukla M, Vonstein V, Wattam AR, Xia F, Stevens R 2014 The SEED and the rapid annotation of microbial genomes using subsystems technology (RAST). Nucleic Acids Res 42:D206–D214. doi:10.1093/nar/gkt1226. 24293654 PMC3965101

[13] Weber T, Blin K, Duddela S, Krug D, Kim HU, Bruccoleri R, Lee SY, Fischbach MA, Müller R, Wohlleben W, Breitling R, Takano E, Medema MH 2015 antiSMASH 3.0—a comprehensive resource for the genome mining of biosynthetic gene clusters. Nucleic Acids Res 43:W237–W243. doi:10.1093/nar/gkv437. 25948579 PMC4489286

[14] Ziemert N, Podell S, Penn K, Badger JH, Allen E, Jensen PR 2012 The natural product domain seeker NaPDoS: a phylogeny based bioinformatic tool to classify secondary metabolite gene diversity. PLoS One 7:e34064. doi:10.1371/journal.pone.0034064. 22479523 PMC3315503

[15] Corretto E, Antonielli L, Sessitsch A, Kidd P, Weyens N, Brader G 2015 Draft genome sequences of 10 *Microbacterium* spp., with emphasis on heavy metal-contaminated environments. Genome Announc 3(3):e00432-00415. doi:10.1128/genomeA.00432-15. 25977426 PMC4432332

[16] Manzo E, Ciavatta ML, Villani G, Varcamonti M, Sayem SM, van Soest R, Gavagnin M 2011 Bioactive terpenes from *Spongia officinalis*. J Nat Prod 74:1241–1247. doi:10.1021/np200226u. 21548580

